# Assessment of the accuracy of digital surgical planning and its implementation with tibial plateau levelling osteotomy

**DOI:** 10.1007/s11259-026-11253-w

**Published:** 2026-05-13

**Authors:** Henry Todd, Jack Fawsitt, Nicholas Goody, Darren James Shaw, Dylan Neil Clements

**Affiliations:** 1 Hospital for Small Animals, Royal (Dick) School of Veterinary Studies and Roslin Institute, Midlothian, EH25 9RG UK; 2 Royal Veterinary College, London, UK

**Keywords:** Tibial plateau levelling osteotomy, Training, Pre-operative planning, 3D printing

## Abstract

**Introduction:**

Pre-operative planning of orthopaedic procedures is widely regarded as critical to their safe execution; however, the ability to both accurately plan and enact the plan have not been assessed in the veterinary context. The aim of this study was to assess the ability of boarded surgeons compared to surgical trainees to reproducibly plan a TPLO and then to implement this plan *in vivo* and *in vitro*.

**Methods:**

Twelve participants planned 10 TPLOs from blinded radiographs of dogs diagnosed with cranial cruciate ligament disease; this was repeated four weeks later and compared between surgical groups using linear mixed-effect models (LME). To assess the ability of a participant to implement their pre-operative planning *in vivo*, pre-operative TPLO plans were compared to post-operative radiographs for each patient using LME in 6 participants. *In vitro*, three 3D printed tibial models were provided to 8 surgeons. Each participant was asked to perform a TPLO osteotomy to predefined measurements and accuracy of this osteotomy was compared between groups using LME

**Results:**

Neither the accuracy of pre-operative planning, nor the ability to accurately implement the plan at surgery, consistently changed with surgeon experience. Using 3D printed models, surgical trainees differed significantly from predetermined D1 (*p* = 0.019), D2 (*p* = 0.002) values and carried out significantly distomedial to proximolateral angled osteotomies (*p* < 0.001). However, surgical trainees and board-certified surgeons were not significantly different from each other when assessing the accuracy of *in vitro* osteotomies.

**Conclusion:**

Pre-operative planning is accurate across differing experience levels and is a valuable tool for the safe execution of canine TPLOs. Accuracy of implementation of the plan during surgery is similar between groups, but surgical trainees were less accurate than residency trained surgeons when undertaking TPLO osteotomies on tibial models. This highlights the critical requirement for intra-operative supervision of surgical trainees.

**Supplementary Information:**

The online version contains supplementary material available at 10.1007/s11259-026-11253-w.

## Introduction

Cranial cruciate ligament disease (CCLD) is the most common cause of stifle lameness in canine patients (Johnson and Johnson 1993). Multiple surgical options exist for surgical management of CCLD with tibial plateau levelling osteotomy (TPLO) providing superior outcomes when compared to tibial tuberosity advancement and extracapsular suture up to 3 years post operatively (Gordon-Evans et al. 2013; Krotscheck et al. 2016; Moore et al. 2020). A survey of both general practitioners and ACVS diplomates revealed TPLO to be the most utilised treatment of CCLD in large dogs in both groups (Duerr et al. 2014).

Pre-operative planning of orthopaedic procedures is essential for their safe execution (Kosashvili et al. 2009; Collins et al. 2014; Holzer et al. 2019) and planning of the TPLO is critical to achieve appropriate and safe positioning of the osteotomy and may increase the likelihood of achieving the planned post-operative tibial plateau angle (TPA) (Kowaleski and McCarthy 2004; Collins et al. 2014). However, the magnitude of the effect of inaccurate osteotomy placement upon post-operative TPA has been demonstrated to be minor in all but the smallest of patients (Mazdarani et al. 2022). Aside from the potential benefit in achieving the desired post-operative TPA, pre-operative planning has been utilised to theoretically correct cases of excessive TPA (Worden et al. 2023; Story et al. 2024), as well as assessing the planned and achieved TPA following cranial closing wedge ostectomy (Banks et al. 2024). Further, there is an increased risk of tibial tuberosity avulsion in large breed dogs following TPLO when the post-osteotomy tibial tuberosity width is less than 10 mm (Bergh et al. 2008); by assessing the patient’s anatomy through pre-operative imaging and utilising pre-operative planning, surgeons may reduce the incidence of iatrogenic tibial tuberosity fractures.

Digital radiography and planning software significantly enhances the precision of TPA measurements by reducing interobserver variability. This method provides consistent results that are independent of the observer’s experience level, potentially demonstrating an improvement over planning using radiographic film (Unis et al. 2010). Despite this, the measured postoperative TPA frequently differs from the pre-operatively planned angle. Objective comparison of postoperative TPA is challenging due to differences in reporting between studies. One study reports over-rotation leading to a smaller than planned TPA (< 4°) in 5/31 cases, appropriate rotation (TPA 4–8°) in 9/31 cases and most frequently, under-rotation (TPA > 8°) in 17/31 cases (Robinson et al. 2006). In contrast Kowaleski reports a more consistent post operative TPA of 4.8±1.9° (Kowaleski et al. 2013). More recently, the use of intraoperative fluoroscopy has been advocated to potentially improve postoperative TPA accuracy with a planned postoperative TPA of 3° and an achieve postoperative median TPA of 3° (range 0–4.5.5°) (Wang et al. 2025).

In the veterinary context, the ability of surgeons of differing experience levels to digitally plan orthopaedic procedures requiring an osteotomy and implant placement, and to accurately enact this plan, have not been assessed. With the variation in size, weight and morphology of veterinary patients, it is inevitable that certain cases will present intra-operative challenges. In the context of TPLO, cases that are marginal between implant sizes and consequently require a larger osteotomy radius to accommodate the larger implant, may require enhanced intra-operative accuracy in order not to violate the joint space or osteotomy with screws, as well as avoiding a post-osteotomy tibial tuberosity width of less than 10 mm. Consequently, this study aimed to assess the ability of 2 groups of surgeons of differing experience – surgeons (DECVS or surgeons post ECVS residency) and surgical trainees (ECVS residents and surgical interns) to:

1. consistently plan a TPLO *in silico*;
2. accurately enact this plan *in vivo;*
3. accurately perform a TPLO osteotomy *in vitro*.

We hypothesised that consistency of *in silic*o TPLO planning, *in viv*o surgical accuracy, and *in vitro* surgical accuracy will all improve in the group with increased surgeon experience.

## Methods

Twelve participants were enrolled on this study from a single university teaching veterinary small animal hospital. Participants were divided into two groups: “SURG” (DECVS or surgeons post ECVS residency) and “TRAINEE” (ECVS residents and surgical interns). Numbers of participants enrolled in each part of this study vary based on case availability and staff changes, participant numbers by group are reported in the individual methods and results sections of this manuscript. All osteotomies, both *in vivo* and *in vitro* were undertaken without the use of a jig.

### Assessment of accuracy of *in silico* pre-operative planning

To assess TPLO planning, participants were provided with 10 medio-lateral radiographs of the affected stifle of dogs that had been diagnosed with CCLD and asked to plan a TPLO with a post-operative target TPA of 5°. All planning was undertaken using a surgical planning software program (vPOP Pro, Shrewsbury, UK). Four weeks later, participants were asked to re-plan the TPLOs from the same radiographs. All radiographs were blinded with a random number (generated in Microsoft Excel using the RANDBETWEEN(1,100) function) and then re-blinded in the same manner for repeat planning, ensuring no reference between planning sessions.

**Fig. 1:**
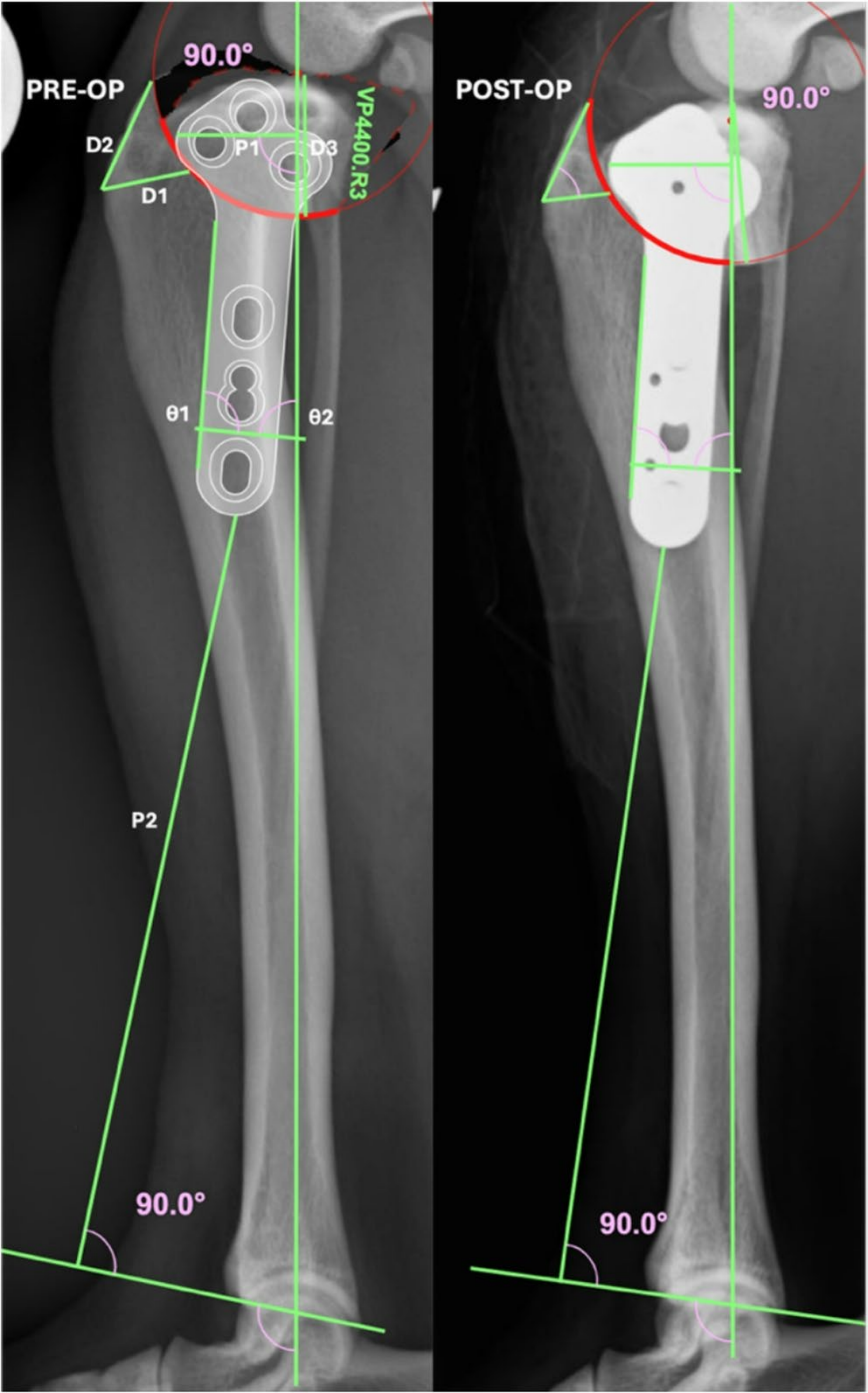
Pre- and post-operative TPLO radiographs of a representative canine hindlimb. Pre-operative planning (left) undertaken with D1, D2, D3, P1 and P2 labelled. Post-operative radiographs have the same parameters marked, but these are unlabelled

If the radius of the osteotomy was different between planning attempts this was recorded and no further measurements are presented. For those radiographs where no change in radius of the osteotomy was recorded, accuracy of osteotomy positioning was assessed using three measurements on the mediolateral radiograph as described by Tan et al. (2014). Briefly; D1 was the distance from the insertion of the patellar tendon to the proposed osteotomy, when measured perpendicular to the cranial aspect of the tibial tuberosity, D2 was the distance from the insertion of the patellar tendon to the proposed osteotomy, when measured along the proximal aspect of the tibia and D3 was the distance from the tibial plateau, at the midpoint of the intercondylar tubercles, measured to the proposed osteotomy at the point where it intersects the caudal tibial cortex, all measured in mm (Tan et al. 2014), as shown by Fig. 1.

From those radiographs where there was no change in the radius of osteotomy recorded, if there was a change in plate size or orientation (i.e. a TPLO plate applied as designed vs. in reverse) this was recorded, and no further measurements are presented. For those radiographs where no changes had been recorded three further measurements were made to assess plate position. These measurements were: P1 (distance in mm from the most cranial aspect of the TPLO plate to the mechanical axis of the tibia), P2 (distance in mm from the most distal aspect of the TPLO plate to the joint orientation line of the distal tibia and the plate angle. The joint orientation line of the distal tibia is represented by a line connecting the distalmost aspect of the cranial and caudal cortices of the tibia (Petazzoni and Jaeger 2008). The joint orientation line was chosen as the distal reference point for P2, rather than the centre of the talus, to compensate for small discrepancies in positioning between radiographic views (Fig. 1). Plate angle is defined as the angle between the long axis of the plate (drawn on the cranial aspect of the TPLO plate) and the mechanical axis of the tibia, with a positive angle being convergent proximally. Plate angle is calculated: $\:Plate\:angle=180-\theta\:1-\theta\:2$ (Fig. 1). Plate orientation refers to intentionally using a left TPLO plate on a right tibia, or vice versa, to facilitate more appropriate plate orientation or to prevent the cranial locking screw in the proximal fragment being excessively close to the osteotomy.

For this *in silico* section 4 SURG (S1-S4) and 7 TRAINEE (T1-T7) provided data. Individual numbers of data points available for each parameter for each participant are visually represented in raster plots in Supplemental Figure S1, depending on whether changes in radius of the osteotomy or plate size and orientation were reported.

### Assessment of *in vivo* accuracy

To assess the ability of participant to accurately enact their pre-operative planning *in vivo*, pre-operative vPOP plans were compared to post-operative radiographs for up to the last 10 consecutive TPLOs. For inclusion the participant must have both made the cresenteric osteotomy and positioned the TPLO plate. Not all participants had 10 available TPLOs for inclusion due to several factors including post-residency duration (post residency surgeons did not use TPLOs that were undertaken during their residency), stage of residency, or clinicians who did not routinely use planning software or save their pre-operative plans. In this *in vivo* section, 3 SURG (S1 *n* = 10, S2 *n* = 5, S3 *n* = 6) and 3 TRAINEES (T2 *n* = 2, T3 *n* = 4, T5 *n* = 4) provided data. Measurements included D1, D2, D3, P1, P2 and the plate angle as described above.

### Assessment of *in vitro* accuracy

**Fig. 2:**
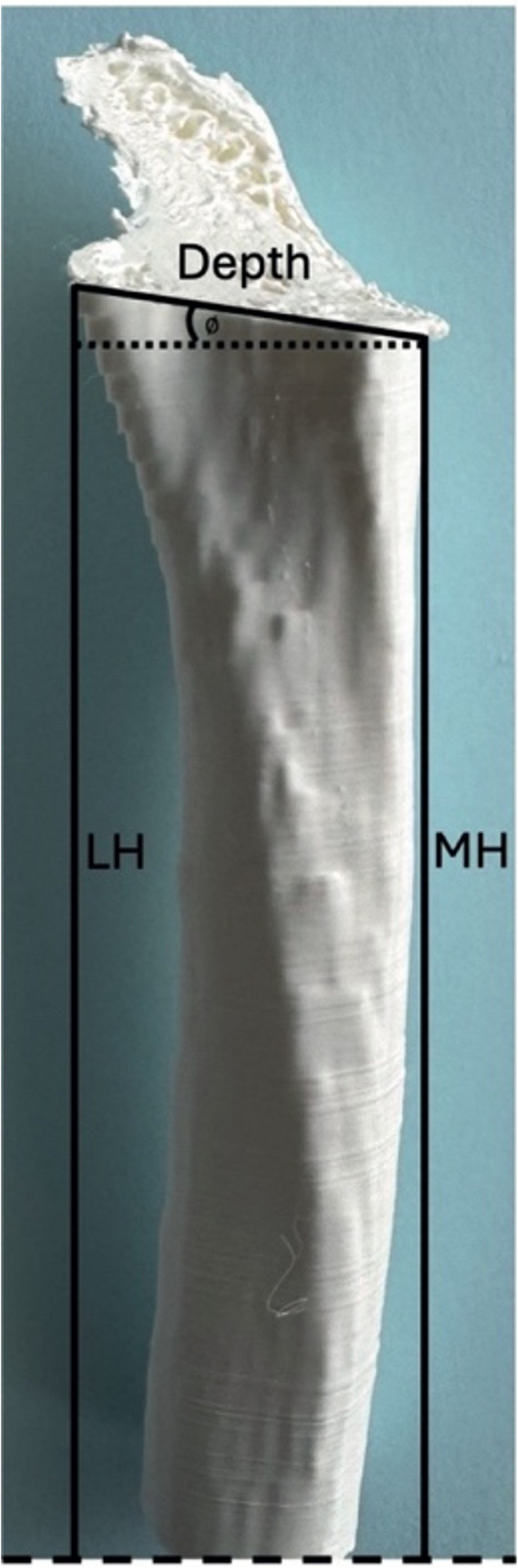
Caudocranial view of a 3D printed tibia following TPLO osteotomy, with the osteotomy angle ($\:{\varnothing}$) and depth of the osteotomy, medial height (MH) and lateral height (LH) indicated. Dashed line at the distal end of the model representing the distal extent of the 3D printed model where measurements were taken to

*In vitro*, three 3D printed tibial models were provided to each participant. A CT scan of the right tibia of a 3-year-old, 37 kg, male, Labrador Retriever was selected for modelling. The length of the right tibia was 191 mm from the proximal intercondylar eminence to the distal epiphysis. Images were acquired using a 64-row multidetector computed tomography scanner (Somatom^®^ Definition AS Siemens, Erlangen, Germany). A selection of DICOM images acquired using a bone kernel (Siemens Br62, 1 mm slice thickness) were exported to 3D image reconstruction software (InVesalius 3, Centro de Tecnologia da Informação Renato Archer, Brazil). A 3D model of the tibia was constructed and was exported as a stereolithography file (.stl). The model was sliced using Prusa Slicer and printed on a Prusa Mini using polylactic acid (PLA) filament with a layer height of 0.15 mm and a 15% infill. The models were printed ‘vertically’ with layer deposition taking place from distal to proximal allowing multiple models to be printed per print run. Osteotomies were undertaken using a 24 mm TPLO saw blade and DeSoutter pneumatic TPLO saw (model DPX-170 S, DeSoutter Medical, Bucks, UK) with oscillation speed of 13,000 cycles per minute. Each participant was instructed to perform a 24 mm TPLO osteotomy to predefined D1, D2 and D3 measurements (D1–10.5 mm, D2–12.8 mm, D3–25 mm), no fragment rotation or fixation with implants was undertaken. Each participant performed the same 24 mm osteotomy on identical models 3 times with a single osteotomy per model. The time taken to perform each osteotomy was not recorded. No attempt to reduce the potential thermal effects of the osteotomy on the 3D printed models (e.g. by cooling or irrigation) was attempted. Achieved measurements of D1, D2, D3 and implant angle were unavailable to each surgeon until all the osteotomies were completed. The 3D printed model was of approximately the proximal 2/3 of the tibia rather than the full tibia. This provided a balance between enough tibial length to facilitate appropriate handling and stabilisation of the ‘bone’ during the osteotomy whist replicating the clinical scenario of not being able to visualise the entire length of the tibia whilst making the osteotomy. The most proximal part of the tibia was obscured with opaque tape (Leukoplast Sleek Latex-Free, Essity, Stockholm, Sweden) to simulate the stifle joint and ensure the requirement to identify the margin of the tibial plateau using a needle as is standard practice during surgery (Alcântara et al. 2025). Accuracy of this osteotomy position was compared between groups, by measuring D1, D2 and D3 with digital callipers and calculating the osteotomy angle as follows: $\:\varnothing\:=\mathrm{sin}\left(\frac{LH-MH}{depth}\right)$ where LH is lateral height of the osteotomy (measured from the distal cut edge of the model) and MH is the medial height of the osteotomy (measured from the distal cut edge of the model), with depth being the deepest part of the osteotomy (at the caudal aspect) as shown by Fig. 2. Given the 3D model was designed in such a way that the mechanical axis of the model represents the mechanical axis of the tibia in the frontal plane, an angle of 0° represents a perpendicular osteotomy. In this *in vitro* section, 3 SURG (S1, S2, S5) and 5 TRAINEE (T1, T2, T3, T5, T7) provided data.

All measurement values represent the mean difference between planning attempts *in silico*, between planned values and those achieve at the time of surgery *in vivo* and between the prescribed osteotomy position and that achieved in vitro, by group. All raw data has been provided in the spreadsheet in Supplemental Information.

### Statistics

Data were analysed in R (v4.5.2, © 2025 The R Foundation for Statistical Computing) and RStudio (v2026.01 build 392, © 2009–2026 Posit Software, PBC). *P* < 0.05 was taken to indicate statistical significance. The packages ggplot2 (v4.0.1), wesanderson (v0.3.7) and gridExtra (v2.3) were used to illustrate the data.

#### *In silico* analyses

Whether the radius of the osteotomy was different between repeat planning, or there was a change in plate size or orientation between repeat planning, or in any of the 3 parameters was assessed using generalised linear mixed effect models with binomial errors (*GLMEb*) using the lme4 (v1.1–35.5.5) and sjPlot (v2.9) packages. To take into account both the use of the same radiograph across participants and that participants evaluated multiple images, both participants and radiographs were entered as random effects, with surgical group (SURG, TRAINEE) entered as a fixed effect. Odds ratios and associated 95% confidence intervals are reported. Least square logit transformed means and associated 95% confidence intervals (CI) from the *GLMEb* were calculated using the package emmeans (v2.01) and back-transformed to percentages.

To assess differences between surgical groups in the difference between planning in TPA, D1-D3, Implant angle, P1 & P2, linear mixed effect models (*LME*) were used. Participants and radiographs were entered as random effects, and surgical group (SURG, TRAINEE) entered as a fixed effect. Residuals were found to be normally distributed. Mean differences associated 95% confidence intervals are reported. Least square means and associated 95% confidence intervals (CI) from the *LME* were then calculated. The number of images and images per participant entered into the LME models are presented in Supplementary Fig. 1.

#### *In vivo* analyses

To assess differences between Surgical groups between what was planned and achieved in D1-D3, Implant angle, P1 & P2, *LME* were used. Participants were entered as random effects, and surgical group (SURG, TRAINEE) entered as a fixed effect. Residuals were found to be normally distributed. Mean differences associated 95% confidence intervals are reported. Least square means and associated 95% confidence intervals (CI) from the *LME* were then calculated. The number of surgeries achieved is indicated above.

#### *In vitro* analyses

To assess differences between surgical groups between the osteotomy they performed on a bone model and the pre-defined value for D1-D3 (10.5, 12.8, 25 mm, respectively) and the osteotomy angle, *LME* were used. Participants were entered as random effects, and surgical group (SURG, TRAINEE) entered as a fixed effect. Residuals were found to be normally distributed. Mean differences associated 95% confidence intervals are reported. Least square means and associated 95% confidence intervals (CI) from the *LME* were then calculated.

## Results

### Assessment of accuracy of *in silico* pre-operative planning

For *in silico* TPLO planning, there was no significant difference (*p* > 0.05) in the frequency that SURG or TRAINEE changed the osteotomy radius, plate size or plate orientation or any of the three between planning attempts (Table 1).

**Table 1:** Least square logit transformed mean percentages and associated 95% confidence intervals (CI), odds ratios (OR and 95%CI) and p-values from the GLMEb statistical models for change in osteotomy radius, implant size and implant orientation for both SURG and TRAINEE groups between *in silico* TPLO planning attempts

	% change + (95% CI)	OR (95%CI)	*P*-value
Change in osteotomy radius	SURG: 19.0% (6.72–43.4) TRAINEE: 30.2% (15.7–50.2)	1.84 (0.43–7.88)	0.410
Change in implant size	SURG: 14.9% (6.8–29.6) TRAINEE: 28.4% (19.0–40.3.0.3)	2.28 (0.83–6.27)	0.112
Change in implant orientation	SURG: 0% TRAINEE: 0.01% (0–43)	N/A	0.965
Any change	SURG: 29.8% (12.7–55.4) TRAINEE: 53.3% (34.4–71.4)	2.69 (0.72–10.15)	0.143

*n* = 4 SURG and *n* = 7 TRAINEE with 10 values per surgeon for all values. N/A no OR possible as no changes in orientation by SURG group

**Fig. 3:**
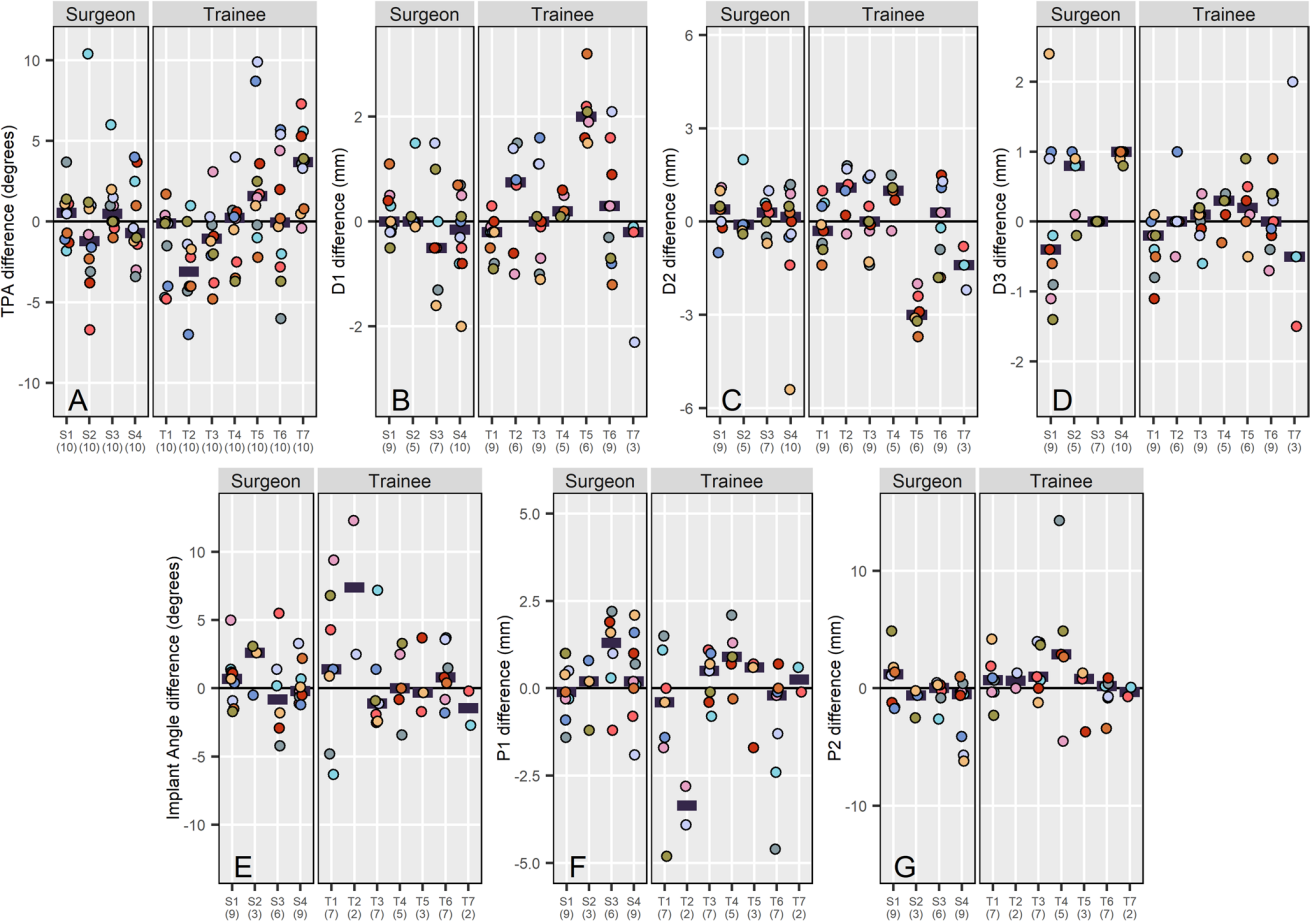
Dot plots showing the difference in (**A**) TPA, (**B**) D1, (**C**) D2, (**D**) D3, (**E**) Implant angle, (**F**) P1 and (**G**) P2 for individual surgeons split by surgical group. Each dot represents the difference in a measured parameter for each case (paired images). Each case is assigned a unique colour. Horizontal bars represent the mean for that participant. The individual number of data points available per surgeon (following data exclusion due to change in osteotomy radius or implant size or orientation) is provided in parentheses

When quantifying the in-silico planning, there was no significant difference (*p* > 0.060) in the change of mean TPA, D1, D2, D3, P1, P2 or implant angle between groups (Tables 1 and 2; Fig. 3).

**Table 2:** Least square mean changes and associated 95% confidence intervals (CI), effect size of difference (β and 95%CI) and p-values from the LME statistical models for TPA, D1, D2, D3, Implant angle, P1 and P2 for both SURG and TRAINEE groups between *in silico* planning attempts

Change in	Mean change + (95%CI)	β (95% CI)	*P*-value
TPA	SURG:$\:\stackrel{-}{\times\:}$= 0.240 (−1.860–2.340) TRAINEE:$\:\stackrel{-}{\times\:}$= 0.081 (−1.540–1.700)	−0.16 (−2.42−2.11)	0.892
D1 *	SURG:$\:\stackrel{-}{\times\:}$= 0.001 (−0.829–0.830) TRAINEE:$\:\stackrel{-}{\times\:}$= 0.309 (−0.327–0.944)	0.31 (−0.60−1.22)	0.205
D2 *	SURG:$\:\stackrel{-}{\times\:}$= 0.101 (−1.130–1.336) TRAINEE:$\:\stackrel{-}{\times\:}$= −0.396 (−1.340–0.552)	−0.50 (−1.85−0.85)	0.465
D3 *	SURG:$\:\stackrel{-}{\times\:}$= 0.366 (−0.043–0.775) TRAINEE:$\:\stackrel{-}{\times\:}$= 0.027 (−0.296–0.350)	−0.34 (−0.78−0.10)	0.061
Implant Angle ^#^	SURG:$\:\stackrel{-}{\times\:}$= 0.525 (−1.208–2.260) TRAINEE:$\:\stackrel{-}{\times\:}$= 0.994 (−0.504–2.490)	0.47 (−1.16−2.10)	0.566
P1 ^#^	SURG:$\:\stackrel{-}{\times\:}$= 0.380 (−0.762–1.522) TRAINEE:$\:\stackrel{-}{\times\:}$= −0.414 (−1.280–0.452)	−0.78 (−1.93−0.39)	0.212
P2 ^#^	SURG:$\:\stackrel{-}{\times\:}$= 0.932 (−1.470–3.330) TRAINEE:$\:\stackrel{-}{\times\:}$= −0.784 (−2.660–1.090)	1.55 (−0.38−3.48)	0.193

* For D1-D3 only the 78 images for which no change in the radius of the osteotomy were analysed (range of images per participant 3–10). ^#^ For Implant angle and P1-P2, only the 60 images for which no change in radius of the osteotomy and/or implant size or orientation were analysed (range of images per participant 2–9). [See Supplementary Fig. 1 for distribution of images between participants]

### Assessment of *in vivo* accuracy

**Fig. 4:**
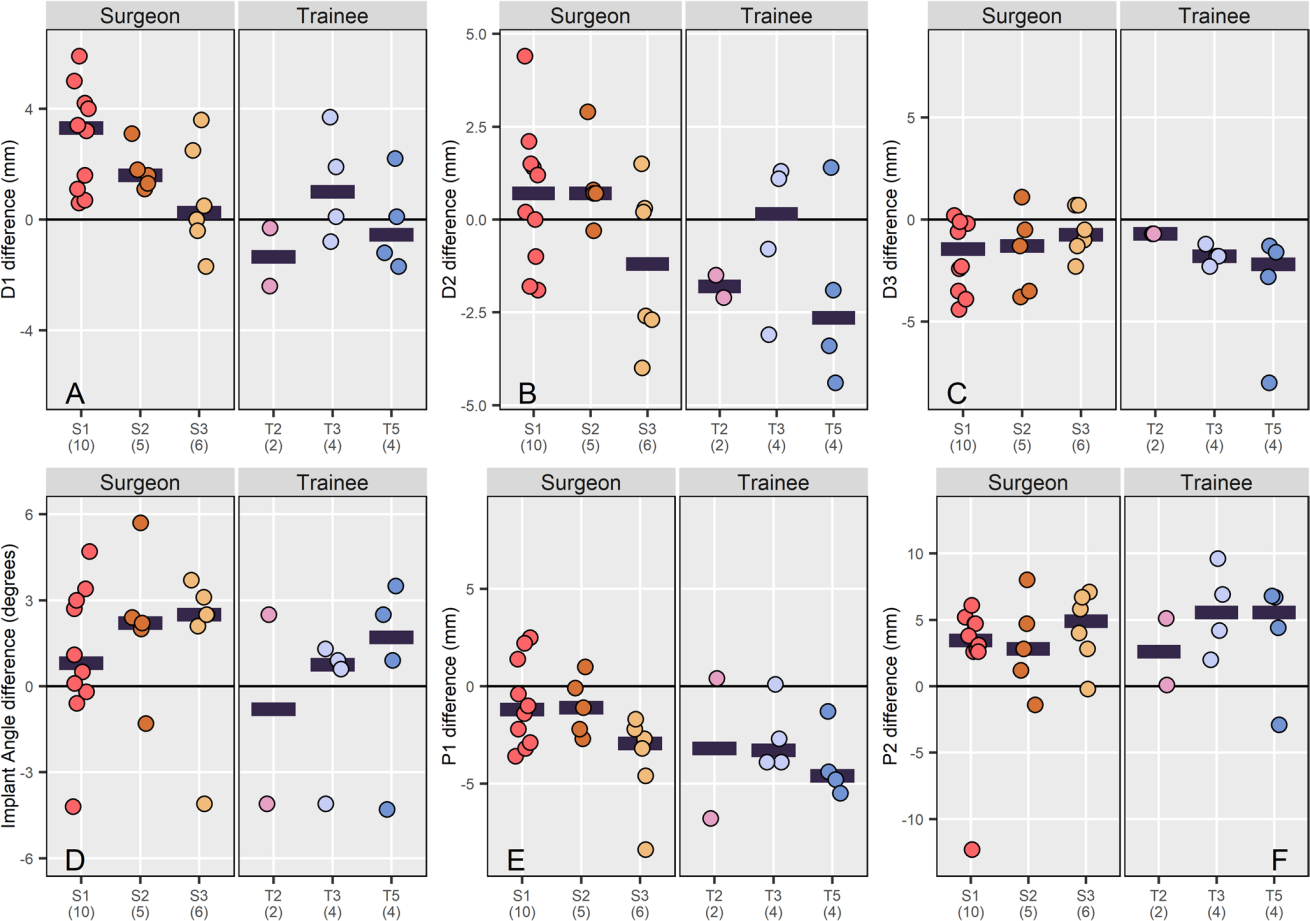
Dot plots showing the difference in (**A**) D1, (**B**) D2, (**C**) D3, (**D**) Implant angle, (**E**) P1 and (**F**) P2 for individual surgeons split by group. Each dot represents the difference in a parameter between planned and achieved values for each case. Each surgeon is assigned a unique colour. Horizontal bars represent the mean for that participant. For all images the individual number of data points available per surgeon (i.e. the number of available TPLOs that met the inclusion criteria) is provided in parentheses

*In vivo*, when assessing the osteotomy position there was no significant difference (*p* > 0.075) in D1, D2, D3, implant angle, P1 or P2 between groups (Table 3; Fig. 4). When assessing D3 both SURG and TRAINEE had a consistently smaller D3 (Fig. 4C) than planned but without a statistically significant difference between groups. Similarly, whilst there was no difference in plate position between groups both groups had a smaller P1 and a larger P2 than planned (Fig. 4E, F).

**Table 3:** Least square mean changes and associated 95% confidence intervals, effect size of difference (β and 95%CI) and p-values from the LME statistical models for D1, D2, D3, Implant angle, P1 and P2 for both SURG and TRAINEE groups between pre-operative planned values and those achieved during surgery *in vivo*. In total 31 surgeries were achieved (range of surgeries per participant 2–10)

Change in	Mean change + (95% CI)	β (95% CI)	*P*-value
D1	SURG:$\:\stackrel{-}{\times\:}$= 1.903 (−0.190–4.000) TRAINEE:$\:\stackrel{-}{\times\:}$= 0.064 (−1.860–1.990)	−1.84 (−3.88−0.20)	0.076
D2	SURG:$\:\stackrel{-}{\times\:}$= 0.137 (−1.930–2.200) TRAINEE:$\:\stackrel{-}{\times\:}$= −1.357 (−3.160–0.440)	−1.49 (−3.40−0.42)	0.122
D3	SURG:$\:\stackrel{-}{\times\:}$= −1.380 (−3.280–0.523) TRAINEE:$\:\stackrel{-}{\times\:}$= −2.210 (−3.600 – −0.821)	−0.83 (−2.33−0.66)	0.262
Implant angle	SURG:$\:\stackrel{-}{\times\:}$= 1.490 (−1.390–4.370) TRAINEE:$\:\stackrel{-}{\times\:}$= −0.030 (−2.000–1.940)	−0.152 (−3.64−0.60)	0.152
P1	SURG:$\:\stackrel{-}{\times\:}$= −1.840 (−4.430–0.741) TRAINEE:$\:\stackrel{-}{\times\:}$= −3.280 (−5.610 - −0.943)	−1.43 (−3.92−1.05)	0.246
P2	SURG:$\:\stackrel{-}{\times\:}$= 3.090 (−1.260–7.430) TRAINEE:$\:\stackrel{-}{\times\:}$= 4.290 (1.320–7.260)	1.20 (−1.99−4.40)	0.447

### Assessment of *in vitro* accuracy

**Fig. 5:**
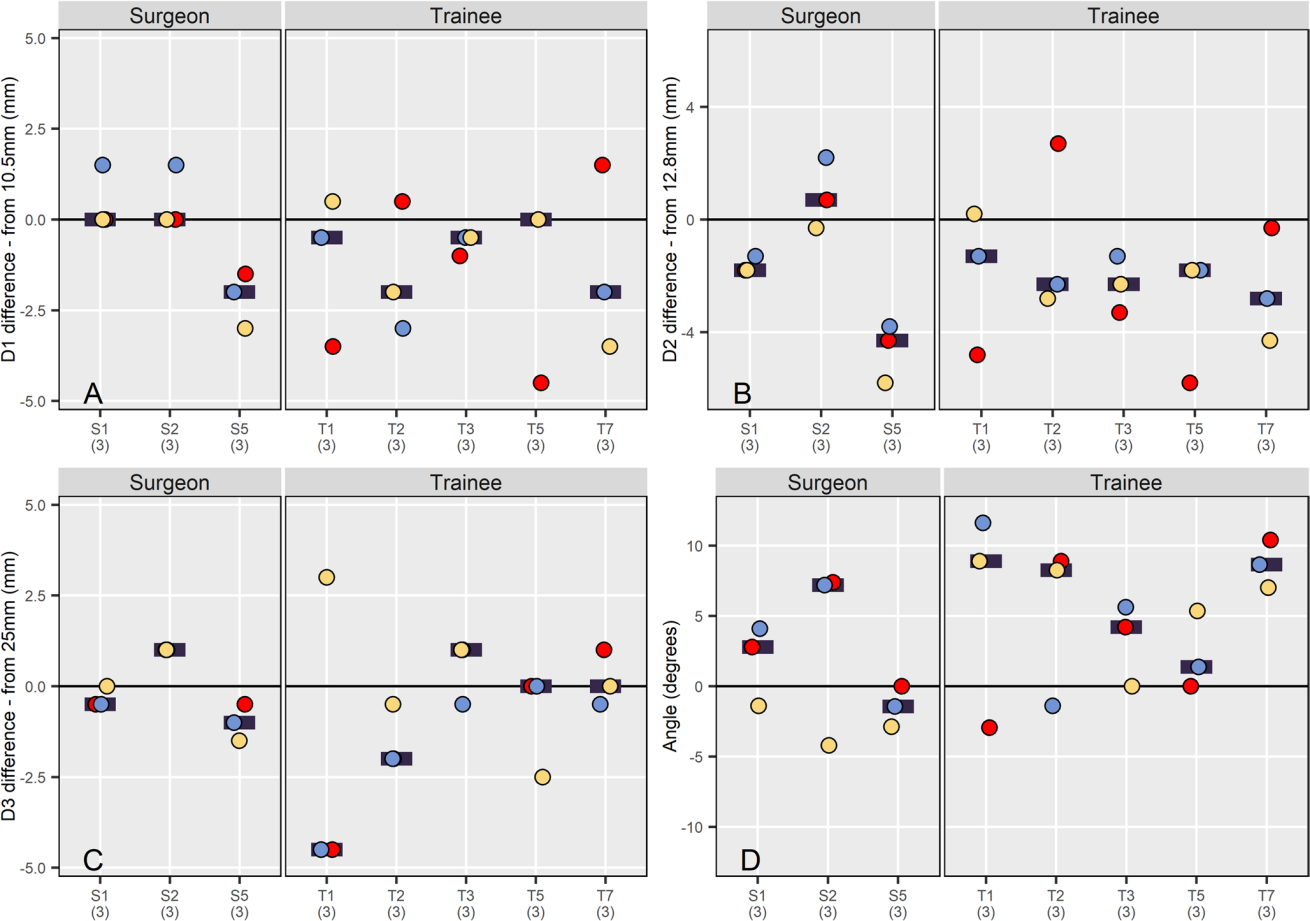
Dot plots showing the difference in (**A**) D1, (**B**) D2, (**C**) D3 and (**D**) Osteotomy angle for individual surgeons split by group. Each dot represents the difference in a parameter between planned and achieved values for osteotomy on a bone model. Each osteotomy attempt is assigned a unique colour 1 st – red, 2nd – blue, 3rd – yellow. Horizontal bars represent the mean for that participant. For all surgeons *n = 3*

*In vitro*, using 3D printed models, there was no significant difference (overall P-value - *p* > 0.061) in accuracy of execution of the osteotomy when measuring D1, D2, D3 or osteotomy angle (Table 4; Fig. 5), where a difference of 0 mm or degrees represents a perfectly accurate and perpendicular osteotomy. When assessing if the mean difference from the planned values for SURG and TRAINEE separately SURG was not significantly different from 0 for any measure (*p* > 0.300), whereas TRAINEE was significantly different from 0 for D1, D2 and osteotomy angle (*p* < 0.018, Table 4).

**Table 4:** Least square mean difference from assigned value (mm of the first cut for D1-3, 0 degrees for Osteotomy angle) and associated 95% CI, effect size (β (95% CI)) and overall P values from the LME statistical models for D1, D2, D3 and osteotomy angles for both SURG and TRAINEE groups during *in vitro* osteotomy of 3D printed canine tibias. Whether individual surgical groups when compared to the assigned value differed from 0 are also presented

Difference from assigned value in	Mean difference from assigned value + (95% CI)	β (95% CI)	Overall*P*-value	*P*-value each group different from 0
D1	Surgeon:$\:\stackrel{-}{\times\:}$= −0.389 (−1.760–0.978) Trainee:$\:\stackrel{-}{\times\:}$= −1.233 (−2.290 – −0.174)	3.77 (−0.19−7.73)	0.246	0.677 **0.019**
D2	Surgeon:$\:\stackrel{-}{\times\:}$= −0.180 (−4.250–0.654) Trainee:$\:\stackrel{-}{\times\:}$= −2.130 (−4.030 – −0.232)	−0.84 (−2.32−0.63)	0.795	0.301 **0.002**
D3	Surgeon:$\:\stackrel{-}{\times\:}$= −0.111 (−1.590–1.371) Trainee:$\:\stackrel{-}{\times\:}$= −0.733 (−1.880–0.415)	−0.33 (−2.98−2.31)	0.426	0.856 0.193
Osteotomy angle	Surgeon:$\:\stackrel{-}{\times\:}$= 1.290 (−2.380–4.960) Trainee:$\:\stackrel{-}{\times\:}$= 5.060 (2.220–7.900)	−0.62 (−2.22−0.98)	0.061	0.406 **< 0.001**

Whilst undertaking the osteotomy the 3D printed tibias remained intact, without overt evidence of warping or fracturing. Osteotomies that were undertaken slowly led to a degree of softening or melting of the model, but the severity of this was mild and was not perceived to have affected the measurements made during this experiment.

## Discussion

Contrary to our hypothesis, neither the accuracy of *in silico* pre-operative planning nor the ability to accurately execute the plan *in vivo* changed markedly with increasing surgeon experience although differences between groups emerged during assessment of *in vitro* osteotomy accuracy.

*In silico*, both residency trained surgeons and trainees had a similar mean difference of typically around 1 mm between planning sessions across all measures of osteotomy position. Plate positioning was slightly more variable with the mean difference between planning sessions being ~2 mm. Residency trained surgeons appeared to position their implants more parallel to the mechanical axis of the tibia than trainees, however, this failed to reach statistical significance. The clinical impact of this is poorly understood, with no consistent biomechanical effect of plate position on construct strength or the magnitude of ‘rockback’ having been consistently demonstrated (Bula 2021; Mclean et al. 2024). Fundamentally, differences in osteotomy position were minimal at both time points in both groups which reflects the ease and formulaic nature of *in silico* planning. The fact that plate position exhibited greater differences in both groups compared to osteotomy position likely reflects the lack of formal guidelines for plate position, as well as a lack of specific measurements, instead focusing on ensuring screws are in bone, and not the osteotomy or articular space.

A key and unexpected result was the frequency with which the osteotomy radius and plate size changed during *in silico* pre-operative TPLO planning by both trainees and residency trained surgeons. Despite the relatively frequent incidence of inconsistent osteotomy size and implant selection from residency trained surgeons, trainees were subjectively more variable than residency trained surgeons (Table 1). The potential impact of this inconsistency from the trainees is that dogs may receive inappropriately sized osteotomies or implants. However, these data demonstrate that even experienced surgeons were also inconsistent in these measures during repeat planning. No patient information (e.g. weight, size, or morphology) was provided during the planning stages; this metadata may guide surgeon osteotomy and plate size selection, based on previous experience and published body weight guidelines. To the authors’ knowledge there are currently no published guidelines regarding TPLO plate selection that take the patient morphology into consideration, instead previous instructions appear to focus solely upon bodyweight (https://www.arthrexvetsystems.com/resources/vLR1-000007-en-US/tplo-plate-system-weight-reference-chart?referringteam=vet_systems___small_animal).

When performing a TPLO *in vivo* there was no significant difference in accuracy of the osteotomy placement between groups with the mean D1, D2 and D3 measures within approximately 2 mm of the pre-operative plan by both residency trained surgeons and trainees. When considering the 0.6 mm thickness of Synthes TPLO saw blades (https://www.movora.co.uk/synthes-tplo-saw-blades), this demonstrates a consistently high level of accuracy. Plate positioning was slightly more variable with both residency trained surgeons and trainees placing the plate within approximately 5 mm of the pre-operative plan. When considering overall trends, rather than solely comparing between groups, residency trained surgeons had a slightly larger D1 than planned, which may be reflective of greater experience of, or “learned” caution to increase tibial tuberosity size of that planned to minimise the risk of, a tibial tuberosity fracture than trainees. Both groups had a smaller than planned D3, which suggests using the intercondylar eminence on the medio-lateral radiograph as the landmark to act as the centre of rotation, may not perfectly align with centre of rotation identified *in vivo* using a needle. The importance of accurate needle positioning along the medial collateral ligament, as well as the stifle being held at an angle 135° has recently been demonstrated in an ex vivo study and may explain these D3 differences (Alcântara et al. 2025).

*In vivo*, both groups have a smaller P1 and a larger P2 than planned. These changes will ensure that the locking screws in the proximal fragment are further from the osteotomy, as the plate is positioned more caudally and proximally, but it is unknown if this is a subconscious decision at the time of surgery, or an unconscious deviation from the plan (i.e. due to the noted increased D1 and reduced D3), as none of the surgeons in this study measured their plate position pre- or intra-operatively to check if it was correct. This may also explain the greater variability in plate position when compared to osteotomy position. The large negative change in P2 for surgeon S1 in Fig. 4 represents a deviation from the pre-operative planned 3.5 mm standard TPLO plate to utilisation of a 3.5 mm broad TPLO plate – this larger implant extending more distally along the tibia with consequent reduction in P2. This represents the only incidence of a deviation in implant size *in vivo*. Finally, both residency trained surgeons and trainees had an increased plate angle, meaning the distal part of the plate was tilted cranially and the proximal part of the plate tilted caudally. This may be a consequence of having a larger than planned D1 (i.e. a more caudal than planned osteotomy, as the natural caution of a surgeon would tend to increase D1 length rather than shorten it, as shortening could increase the risk of tibial tuberosity fracture) and thus a smaller P1 to ensure the screws in the distal fragment were appropriately centred on the tibial diaphysis. The clinical impact of this deviation from the pre-operative plan is challenging to assess, especially given the inconsistencies in size and morphology of veterinary patients. Regardless, with such small absolute differences, the clinical consequence may be negligible.

**Fig. 6:**
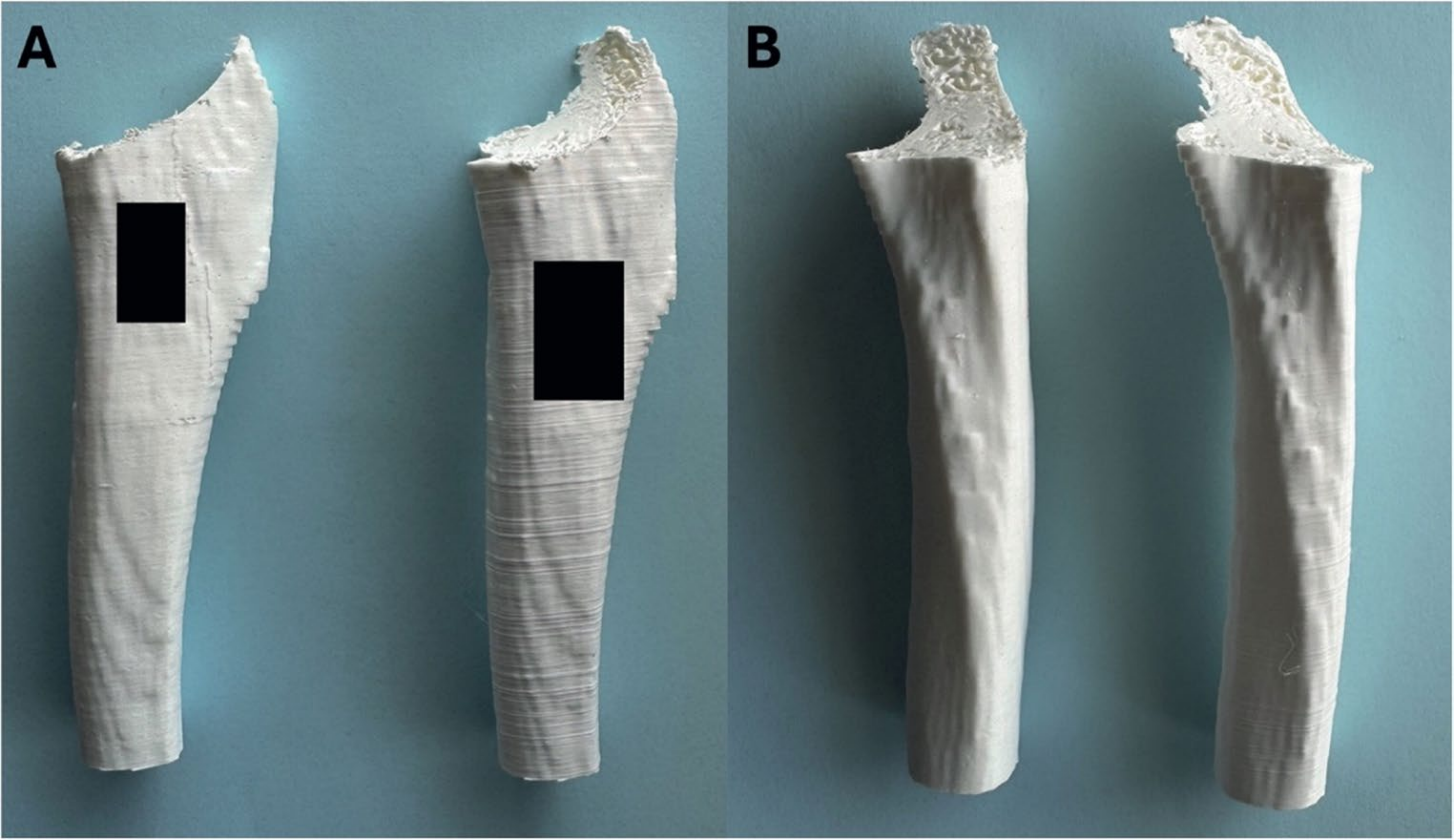
Lateromedial (**A**) and caudocranial (**B**) views of 2 3D printed tibias following TPLO osteotomy demonstrating a visible difference in osteotomy angle (LEFT tibia 0° vs. RIGHT tibia 10.4°). Black squares hiding surgeon and sample name

To investigate whether the consistent accuracy of *in vivo* TPLO execution was due to a lack of difference in technical ability, or adequate intra-operative supervision, unsupervised *in vitro* osteotomies were undertaken on 3D printed models of canine tibias. This again showed no difference between groups for accuracy of D1, D2 or D3. When comparing the osteotomy angle, trainees subjectively made a more distomedial to proximolateral angled osteotomy than residency trained surgeons; with angles ranging from 0° to 10.4° (Fig. 6); however, this degree of angulation is unlikely to be clinically significant (Wheeler et al. 2003).

However, when assessing if there was a difference between the planned position and angle of the osteotomy and that which was achieved *in vitro*, surgical trainees were significantly different from the planned values for D1, D2 and osteotomy angle, demonstrating that surgical trainees were less accurate than residency trained surgeons *in vitro* and allowing us to accept the final part of our hypothesis. That this difference is present *in vitro* and not *in vivo* may be explained by adequate intra-operative supervision and a consequent inability to significantly deviate from the pre-operative plan.

Whilst the use of a TPLO jig is frequently advocated, there is conflicting evidence to support them facilitating more accurate osteotomies (Bell and Ness 2007; Tan et al. 2014; Craig et al. 2017). Furthermore, Craig et al. (2017) demonstrate a non-significant improvement in the accuracy of osteotomy positioning with increasing surgeon experience without the use of an osteotomy guide, showing some similarity to the data presented in this manuscript. More recently the use of custom 3D printed osteotomy guides has been demonstrated to consistently improve accuracy of TPLO osteotomies (Kim et al. 2021). Whilst the effect of eccentric osteotomy position on post-operative TPA is small (Mazdarani et al. 2022), the clinical impact of an inaccurately positioned osteotomy may be much more consequential, for example, if this results in a screw being placed in the osteotomy or joint, or, with a D1 of less than 10 mm (Bergh et al. 2008).

That we have demonstrated similar accuracy between SURG and TRAINEE groups *in silico* and *in vivo* may be due to several factors. Thorough training of residents in the planning and execution of a TPLO, critically, by those surgeons enrolled in the SURG group, will undoubtedly have contributed to similar accuracy between groups. This, coupled with the formulaic nature of TPLO planning and ease of use of commercially available software, explains the remarkably consistent *in silico* results. *In vivo*, residents were always supervised by senior surgeons, preventing gross deviation from the pre-operative plan. Finally, the lack of clinical consequence from error, *in silico* and *in vitro*, may result in less attention to detail than would usually be applied to clinical cases; conversely, the fact that individual surgeons planning will be scrutinised by a third party may lead to greater attention to detail.

This study has several limitations, including the small number of surgeons involved and the relatively small number of clinical cases/repeats. The TRAINEE group was an amalgamation of both ECVS residents and surgical interns. Stratifying the enrolled surgeons into resident and intern groups may have provided further information regarding experience level, but with such low numbers enrolled in the study, any additional information would be challenging to interpret. Collecting further clinical cases to use would have been possible for residency trained surgeons; however, the surgical trainees submitted all available cases that met the criteria of the trainee undertaking both the osteotomy and plate positioning.

This study was underpowered to detect small differences; However, for completeness statistical analysis has been included throughout this manuscript. Undertaking a multi-centre study to ensure adequate enrolment would be of benefit in the future, and this study may act as a useful starting point, noting that the centre is likely to have an effect due to differences in residency training.

During *in silico* repeat planning of TPLOs, no comparison of the absolute size of D1, D2 and D3 between surgical trainees and residency trained surgeons was undertaken, instead only the change in these measures was assessed. This was due to different sizes and morphologies of dogs being used for the planning, making assessment of the magnitude of change less useful between groups given the range of patient sizes from toy to giant breed. This patient selection was intentional, given that it is clinically imperative that surgeons are capable of accurately planning TPLOs for all patient sizes.

Further limitations include the method of measuring P1 and plate angle, as the mechanical axis of the tibia has the potential to shift with an eccentric osteotomy (Kowaleski et al. 2005). The likelihood of this is small, and consequently, this remained the most consistent way of assessing these measures. Whilst measuring P1 relative to the tibial tuberosity is possible (assuming perfect compression of the osteotomy in the cranio-caudal direction), there was no viable alternative for measuring plate angle.

Finally, the use of 3D printed tibias whilst cost effective and ethical, is not mechanically identical to using fresh, cadaveric bone. The yield strain of PLA is 60 MPa (Travieso-Rodriguez et al. 2019), whereas the yield strain of human femoral cortical bone has a median value of 112.5 MPa (Baleani et al. 2024). This difference in yield strain, and logically therefore, elasticity, may impact tactile feedback whilst completing the *in vitro* osteotomy and will not perfectly replicate canine tibial bone. It was noticed in cases where the osteotomy was undertaken slowly that the 3D printed model softened due to thermal damage. If 3D printed models are to be used for similar purposes in the future the use of cooling techniques such water irrigation should be considered. However, no overt deformation of the 3D printed models was noted, meaning the softening of the PLA was unlikely to affect the measurements in this study. Finally, the lack of other anatomic reference points in this model may have made it more challenging to position the osteotomy accurately. Despite these issues, the impact of this on the accuracy of the osteotomy and subsequent measurements was perceived to be small.

In conclusion, surgeon experience had no effect on the accuracy of *in silico* TPLO pre-operative planning nor accuracy of execution of the surgical plan *in vivo. In vitro* there was no significant difference in accuracy between groups, however surgical trainees were significantly less accurate with. Osteotomy position than residency trainees. This study highlights the relative ease and value of pre-operative planning for both inexperienced and experienced surgeons in accurately enacting the pre-operative plan *in vivo* whilst demonstrating the importance of adequate intra-operative supervision.

## Supplementary Information

Below is the link to the electronic supplementary material.

ESM 1 PDF (150 KB)

ESM 2 XLSX (36.7 KB)

## Data Availability

The datasets generated and analysed during the current study are available in the Supplemental Information.
