# Supplementary material for: Assessment of the accuracy of digital surgical planning and its implementation with tibial plateau levelling osteotomy
Source: Vet Res Commun. 2026 May 13;50(4):321. doi: 10.1007/s11259-026-11253-w (PMC13171988; doi:10.1007/s11259-026-11253-w)
Supplement: Supplementary file 1 — PDF (150 KB) [file 11259_2026_11253_MOESM1_ESM.pdf]

Supplementary Figure 1

Assessment of the accuracy of digital surgical planning and its implementation with tibial plateau levelling osteotomy.

Henry Todd* BVSc MSc PhD MRCVS, Jack Fawsitt BSc(Hons) BVetMed AFHEA MRCVS, Nicholas Goody BVM&S MRCVS, Darren James Shaw BSc PhD FSRB, Dylan Neil Clements BSc BVSc PhD DSAS(Orth) DipECVS SFHEA FRCVS.

Hospital for Small Animals, Royal (Dick) School of Veterinary Studies and Roslin Institute, Midlothian, EH25 9RG, UK

* Corresponding author: Henry.Todd@ed.ac.uk

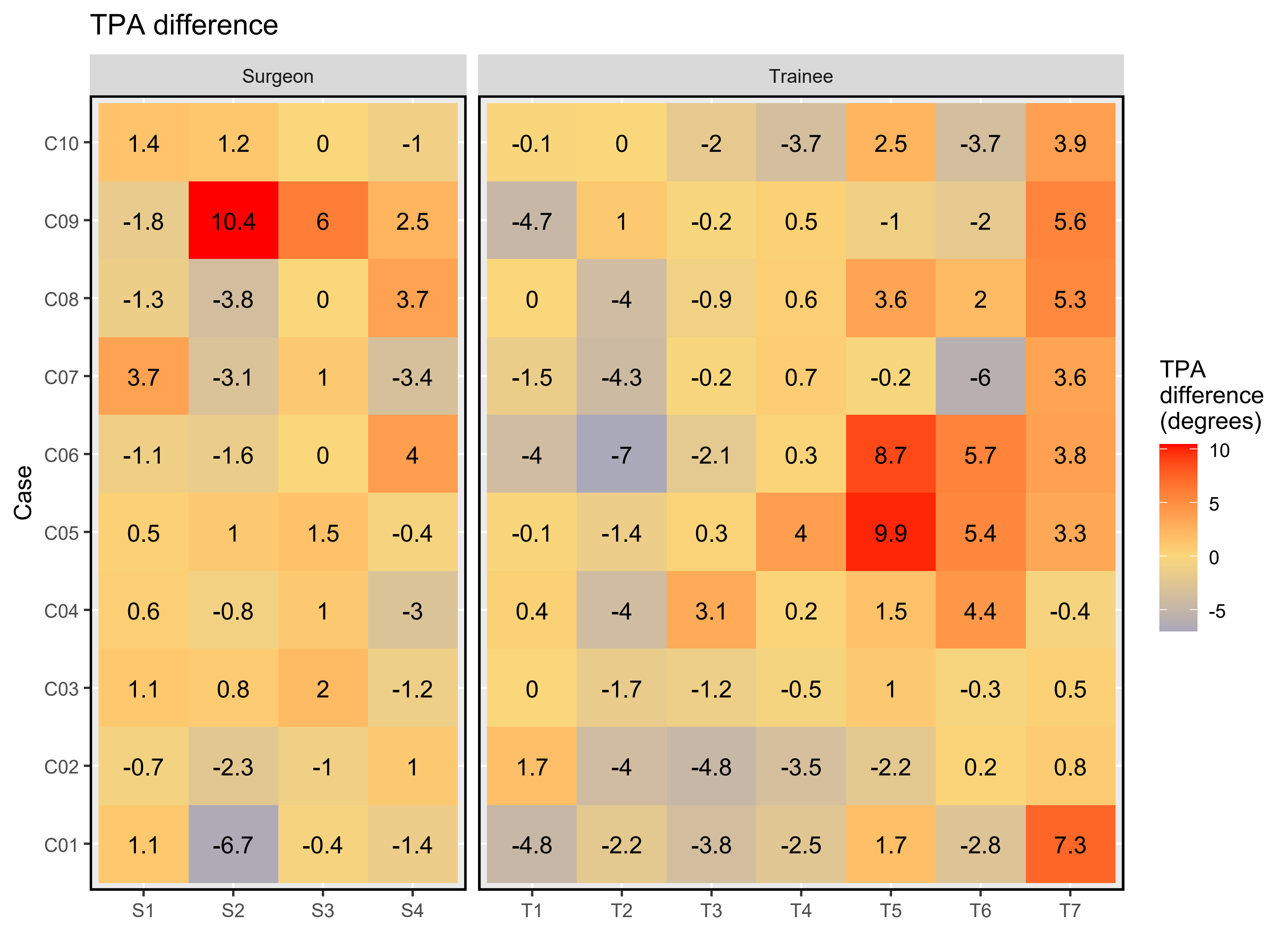

TPA difference
Surgeon
Trainee
Case
C10 1.4 1.2 0 -1 -0.1 0 -2 -3.7 2.5 -3.7 3.9
C09 -1.8 10.4 6 2.5 -4.7 1 -0.2 0.5 -1 -2 5.6
C08 -1.3 -3.8 0 3.7 0 -4 -0.9 0.6 3.6 2 5.3
C07 3.7 -3.1 1 -3.4 -1.5 -4.3 -0.2 0.7 -0.2 -6 3.6
C06 -1.1 -1.6 0 4 -4 -7 -2.1 0.3 8.7 5.7 3.8
C05 0.5 1 1.5 -0.4 -0.1 -1.4 0.3 4 9.9 5.4 3.3
C04 0.6 -0.8 1 -3 0.4 -4 3.1 0.2 1.5 4.4 -0.4
C03 1.1 0.8 2 -1.2 0 -1.7 -1.2 -0.5 1 -0.3 0.5
C02 -0.7 -2.3 -1 1 1.7 -4 -4.8 -3.5 -2.2 0.2 0.8
C01 1.1 -6.7 -0.4 -1.4 -4.8 -2.2 -3.8 -2.5 1.7 -2.8 7.3
S1 S2 S3 S4 T1 T2 T3 T4 T5 T6 T7
TPA difference (degrees)
10
5
0
-5

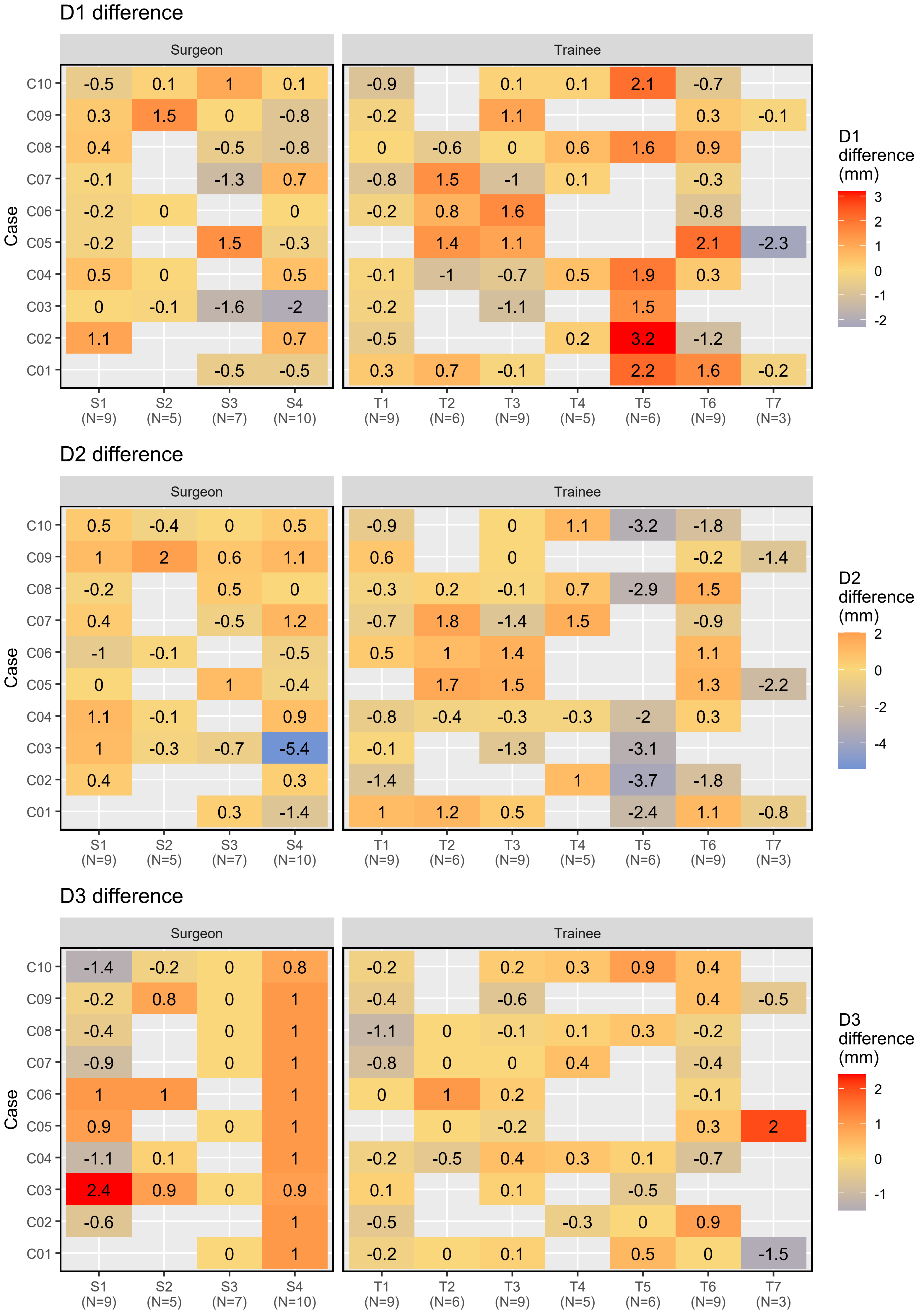
D1 difference
Surgeon
Trainee
Case
C10 -0.5 0.1 1 0.1
C09 0.3 1.5 0 -0.8
C08 0.4 -0.5 -0.8
C07 -0.1 -1.3 0.7
C06 -0.2 0 0
C05 -0.2 1.5 -0.3
C04 0.5 0 0.5
C03 0 -0.1 -1.6 -2
C02 1.1 0.7
C01 -0.5 -0.5
C10 -0.9 0.1 0.1 2.1 -0.7
C09 -0.2 1.1 0.3 -0.1
C08 0 -0.6 0 0.6 1.6 0.9
C07 -0.8 1.5 -1 0.1 -0.3
C06 -0.2 0.8 1.6 -0.8
C05 1.4 1.1 2.1 -2.3
C04 -0.1 -1 -0.7 0.5 1.9 0.3
C03 -0.2 -1.1 1.5
C02 -0.5 0.2 3.2 -1.2
C01 0.3 0.7 -0.1 2.2 1.6 -0.2
S1 (N=9) S2 (N=5) S3 (N=7) S4 (N=10)
T1 (N=9) T2 (N=6) T3 (N=9) T4 (N=5) T5 (N=6) T6 (N=9) T7 (N=3)
D1 difference (mm)
3 2 1 0 -1 -2
D2 difference
Surgeon
Trainee
Case
C10 0.5 -0.4 0 0.5
C09 1 2 0.6 1.1
C08 -0.2 0.5 0
C07 0.4 -0.5 1.2
C06 -1 -0.1 -0.5
C05 0 1 -0.4
C04 1.1 -0.1 0.9
C03 1 -0.3 -0.7 -5.4
C02 0.4 0.3
C01 0.3 -1.4
C10 -0.9 0 1.1 -3.2 -1.8
C09 0.6 0 -0.2 -1.4
C08 -0.3 0.2 -0.1 0.7 -2.9 1.5
C07 -0.7 1.8 -1.4 1.5 -0.9
C06 0.5 1 1.4 1.1
C05 1.7 1.5 1.3 -2.2
C04 -0.8 -0.4 -0.3 -0.3 -2 0.3
C03 -0.1 -1.3 -3.1
C02 -1.4 1 -3.7 -1.8
C01 1 1.2 0.5 -2.4 1.1 -0.8
S1 (N=9) S2 (N=5) S3 (N=7) S4 (N=10)
T1 (N=9) T2 (N=6) T3 (N=9) T4 (N=5) T5 (N=6) T6 (N=9) T7 (N=3)
D2 difference (mm)
2 0 -2 -4
D3 difference
Surgeon
Trainee
Case
C10 -1.4 -0.2 0 0.8
C09 -0.2 0.8 0 1
C08 -0.4 0 1
C07 -0.9 0 1
C06 1 1 1
C05 0.9 0 1
C04 -1.1 0.1 1
C03 2.4 0.9 0 0.9
C02 -0.6 1
C01 0 1
C10 -0.2 0.2 0.3 0.9 0.4
C09 -0.4 -0.6 0.4 -0.5
C08 -1.1 0 -0.1 0.1 0.3 -0.2
C07 -0.8 0 0 0.4 -0.4
C06 0 1 0.2 -0.1
C05 0 -0.2 0.3 2
C04 -0.2 -0.5 0.4 0.3 0.1 -0.7
C03 0.1 0.1 -0.5
C02 -0.5 -0.3 0 0.9
C01 -0.2 0 0.1 0.5 0 -1.5
S1 (N=9) S2 (N=5) S3 (N=7) S4 (N=10)
T1 (N=9) T2 (N=6) T3 (N=9) T4 (N=5) T5 (N=6) T6 (N=9) T7 (N=3)
D3 difference (mm)
2 1 0 -1

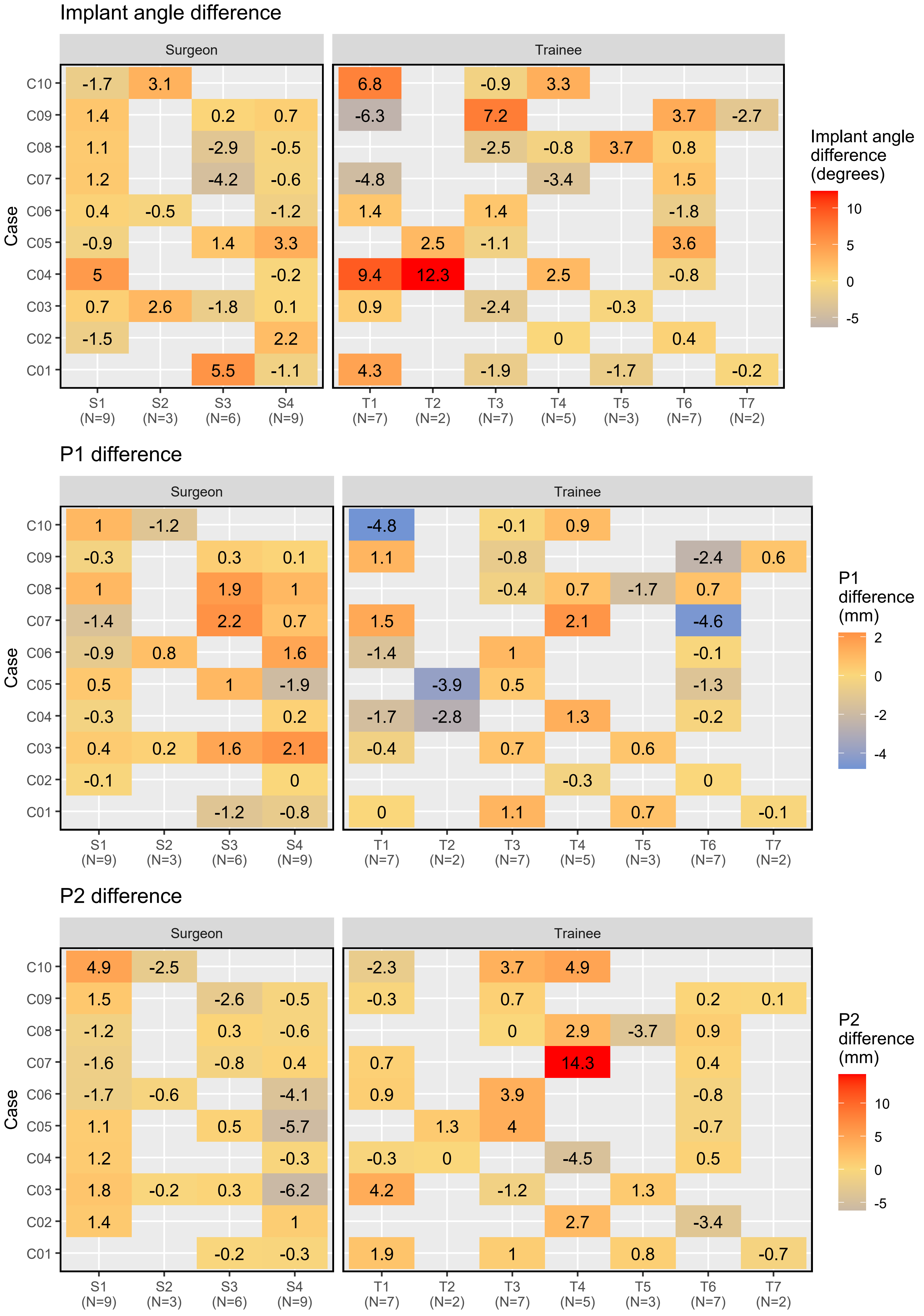
Implant angle difference
Surgeon
Trainee
Case
C10 -1.7 3.1 6.8 -0.9 3.3
C09 1.4 0.2 0.7 -6.3 7.2 3.7 -2.7
C08 1.1 -2.9 -0.5 -2.5 -0.8 3.7 0.8
C07 1.2 -4.2 -0.6 -4.8 -3.4 1.5
C06 0.4 -0.5 -1.2 1.4 1.4 -1.8
C05 -0.9 1.4 3.3 2.5 -1.1 3.6
C04 5 -0.2 9.4 12.3 2.5 -0.8
C03 0.7 2.6 -1.8 0.1 0.9 -2.4 -0.3
C02 -1.5 2.2 0 0.4
C01 5.5 -1.1 4.3 -1.9 -1.7 -0.2
S1 (N=9) S2 (N=3) S3 (N=6) S4 (N=9)
T1 (N=7) T2 (N=2) T3 (N=7) T4 (N=5) T5 (N=3) T6 (N=7) T7 (N=2)
Implant angle difference (degrees)
10 5 0 -5
P1 difference
Surgeon
Trainee
Case
C10 1 -1.2 -4.8 -0.1 0.9
C09 -0.3 0.3 0.1 1.1 -0.8 -2.4 0.6
C08 1 1.9 1 -0.4 0.7 -1.7 0.7
C07 -1.4 2.2 0.7 1.5 2.1 -4.6
C06 -0.9 0.8 1.6 -1.4 1 -0.1
C05 0.5 1 -1.9 -3.9 0.5 -1.3
C04 -0.3 0.2 -1.7 -2.8 1.3 -0.2
C03 0.4 0.2 1.6 2.1 -0.4 0.7 0.6
C02 -0.1 0 -0.3 0
C01 -1.2 -0.8 0 1.1 0.7 -0.1
S1 (N=9) S2 (N=3) S3 (N=6) S4 (N=9)
T1 (N=7) T2 (N=2) T3 (N=7) T4 (N=5) T5 (N=3) T6 (N=7) T7 (N=2)
P1 difference (mm)
2 0 -2 -4
P2 difference
Surgeon
Trainee
Case
C10 4.9 -2.5 -2.3 3.7 4.9
C09 1.5 -2.6 -0.5 -0.3 0.7 0.2 0.1
C08 -1.2 0.3 -0.6 0 2.9 -3.7 0.9
C07 -1.6 -0.8 0.4 0.7 14.3 0.4
C06 -1.7 -0.6 -4.1 0.9 3.9 -0.8
C05 1.1 0.5 -5.7 1.3 4 -0.7
C04 1.2 -0.3 -0.3 0 -4.5 0.5
C03 1.8 -0.2 0.3 -6.2 4.2 -1.2 1.3
C02 1.4 1 2.7 -3.4
C01 -0.2 -0.3 1.9 1 0.8 -0.7
S1 (N=9) S2 (N=3) S3 (N=6) S4 (N=9)
T1 (N=7) T2 (N=2) T3 (N=7) T4 (N=5) T5 (N=3) T6 (N=7) T7 (N=2)
P2 difference (mm)
10 5 0 -5
